# Resonant Transparency and Non-Trivial Non-Radiating Excitations in Toroidal Metamaterials

**DOI:** 10.1038/srep02967

**Published:** 2013-10-17

**Authors:** V. A. Fedotov, A. V. Rogacheva, V. Savinov, D. P. Tsai, N. I. Zheludev

**Affiliations:** 1 Optoelectronics Research Centre and Centre for Photonic Metamaterials, University of Southampton, SO17 1BJ, UK; 2 Department of Physics, National Taiwan University, Taipei 10617, Taiwan; 3 Research Center for Applied Sciences, Academia Sinica, Taipei 115, Taiwan; 4 Centre for Disruptive Photonics Technologies, Nanyang Technological University, 637371 Singapore

## Abstract

Engaging strongly resonant interactions allows dramatic enhancement of functionalities of many electromagnetic devices. However, resonances can be dampened by Joule and radiation losses. While in many cases Joule losses may be minimized by the choice of constituting materials, controlling radiation losses is often a bigger problem. Recent solutions include the use of coupled radiant and sub-radiant modes yielding narrow asymmetric Fano resonances in a wide range of systems, from defect states in photonic crystals and optical waveguides with mesoscopic ring resonators to nanoscale plasmonic and metamaterial systems exhibiting interference effects akin to electromagnetically-induced transparency. Here we demonstrate theoretically and confirm experimentally a new mechanism of resonant electromagnetic transparency, which yields very narrow isolated symmetric Lorentzian transmission lines in toroidal metamaterials. It exploits the long sought non-trivial non-radiating charge-current excitation based on interfering electric and toroidal dipoles that was first proposed by Afanasiev and Stepanovsky in [J. Phys. A Math. Gen. 28, 4565 (1995)].

Toroidal dipole (and higher toroidal multipoles) is not a part of the standard multipole expansion that is usually presented as a sum of contributions from two families of conventional elementary radiation sources, namely the magnetic and electric dynamic multipoles^1^,^2^. In a spherical coordinate system, magnetic multipoles are defined by transversal components of oscillating current density (e.g. currents flowing on a surface of a sphere), while electric multipoles are attributed to oscillating charge density. Fields of an arbitrary charge-current configuration also contain a contribution from oscillating *radial* components of the current density, which is, however, dropped in the commonly used long-wavelength approximation rendering the standard multipole set *incomplete*, as was pointed out in Refs. 3,4,5. These radial components of the current density give rise to an additional independent family of elementary radiation sources, the dynamic toroidal multipoles, which are physically rather than just semantically different from the conventional magnetic and electric multipoles^6^,^7^. While electric and magnetic dipoles can be visualised as a pair of opposite charges and a circular current respectively, toroidal dipole results from currents **j** flowing on the surface of a torus along its meridians (poloidal currents), as illustrated in Fig. 1a. Its moment is given by 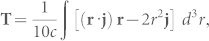
where **r** is the coordinate vector with its origin placed in the center of the torus^3^.

In physics of nuclei static toroidal moment is a well-established notion, also referred to as the anapole. It has been calculated for a number of nuclear systems and investigated in details theoretically and experimentally, in particular, for its role in nuclear-spin-dependent atomic parity violation (see^8^,^9^ and references therein). Toroidal moment has also been identified from first principle calculations in carbon macromolecules^10^ and ferroelectric nanostructures^11^. However, the dynamic excitations of toroidal dipole and higher toroidal multipoles were overlooked for some time in classical electrodynamics as their manifestations are often masked by much stronger electric and magnetic multipoles. Only recently, a spectrally isolated transmission resonance corresponding to the toroidal excitation has been observed in an artificially engineered electromagnetic material (metamaterial), where contributions from electric and magnetic multipoles were deliberately suppressed by design^12^. Since then microwave and optical toroidal resonances were identified in a number of metamaterial and plasmonic systems^13^,^14^,^15^,^16^.

Importantly, the emissions of oscillating electric and toroidal dipoles have the same angular distribution and parity properties, although there are fundamental physical differences between the two elementary excitations: (i) radiated power for electric and toroidal dipoles scales as correspondingly^5^,^6^,^7^ *ω*^4^ and *ω*^6^; (ii) electric dipole is induced by the electric field of the wave, **E**, while toroidal dipole is induced by^12^ **curl** **B** ~ d**E**/d*t*; (iii) vector potential fields **A** generated by oscillating electric and toroidal dipoles are different and the difference cannot be removed by gauge fixing^17^. Thus, when the poloidal current mode (toroidal dipole) and bipolar charge distribution (electric dipole) are collocated and coherently oscillate at the same frequency, their radiated electromagnetic fields can be made to interfere destructively and disappear everywhere outside the resulting charge-current configuration, as was first pointed out by Afanasiev and Stepanovsky^17^ and demonstrated with further numerical studies^18^. This gives an interesting opportunity of creating artificial electromagnetic materials, which exhibit a very narrow isolated transparency band when the radiation losses are suppressed through the interference of toroidal and electric dipolar emission. The underpinning mechanism of the transparency and resonant dispersion here are somewhat different from those typically encountered in the plasmonic and metamaterial systems mimicking electromagnetically-induced transparency, where the interference between two spectrally separated multipolar modes produces a sharp asymmetric Fano resonance on the background of another much wider resonant band^19^.

## Results

**Figure 1:**
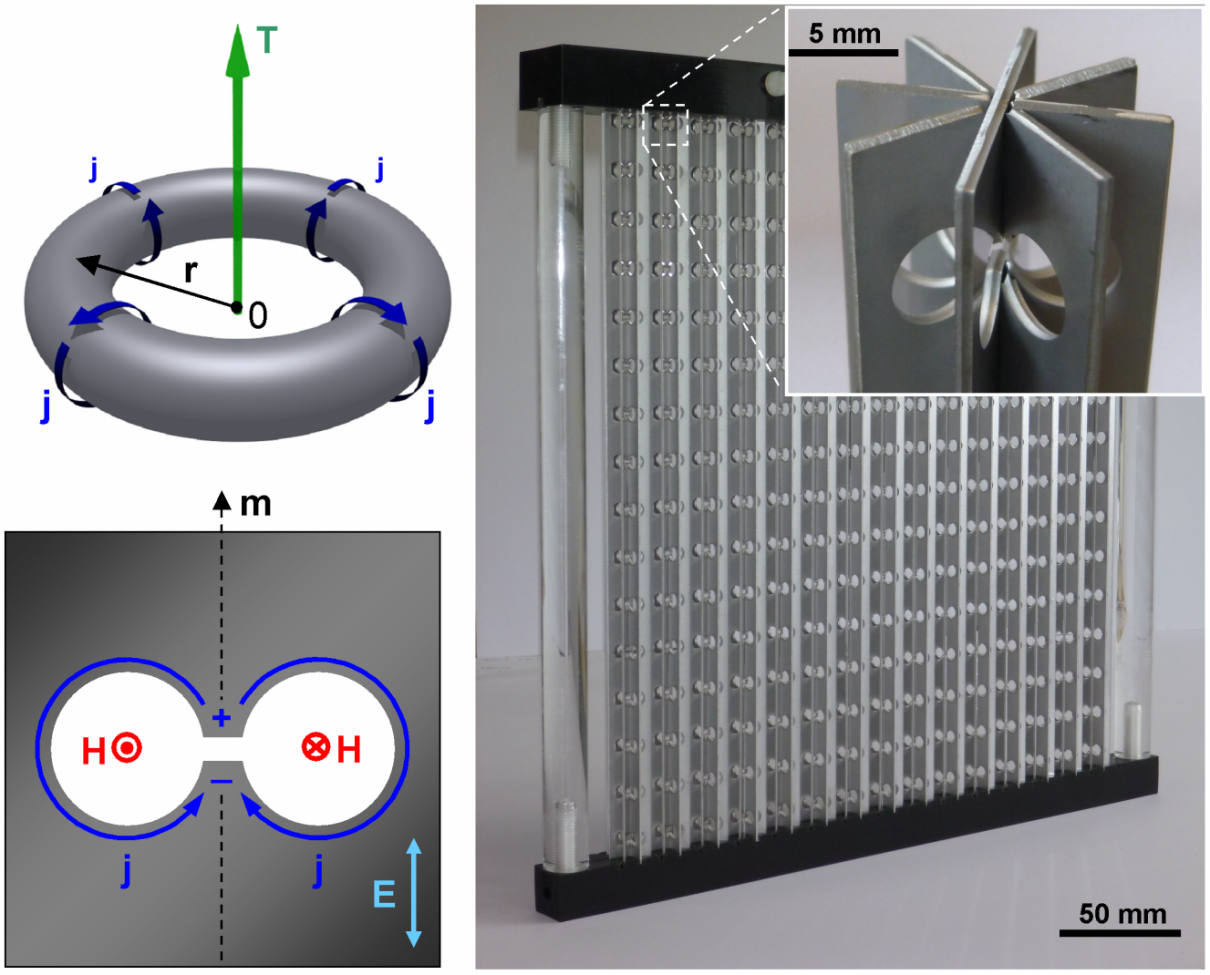
Toroidal metamaterial. (a) Poloidal currents flowing on a surface of a torus along its meridians create toroidal dipole moment **T**. (b) Metal screen with a dumbbell-shaped aperture is the structural element of toroidal metamaterial. Dashed arrow **m** represents axis of its mirror symmetry. (c) Photograph of the assembled metamaterial slab, an array of 15 × 16 toroidal aperture-based metamolecules. Inset shows a close-up view of one of the array's column with 8-fold symmetry.

We demonstrate this novel mechanism of radiation suppression for metamaterials based on a dumbbell-shaped aperture element. It is made in a thin metal plate and resembles a meridianal cross-section of a toroidal void (see Fig. 1b). This is a very special electromagnetic system in which the incident wave **E**(*t*) = **E**_0_ e^i*ωt*^ polarized parallel to the symmetry axis of the aperture **m** induces both electric **P** and toroidal **T** dipolar moments. Indeed, the electric field of the wave drives charge separation *ρ* (*t*) = *ρ*_0_ e^i*ωt*^ across the waist of the aperture, which gives rise to an oscillating electric dipole moment 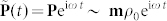 oriented along **m** (see Fig. 1b). The charge displacement also gives rise to counter-rotating (poloidal-like) currents *j*(*t*) oscillating along the edges of the circular cuts, as shown in Fig. 1b. The poloidal currents are proportional to the time derivative of the charge displacement and produce toroidal moment 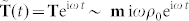 that is also oriented along **m** and oscillates coherently with the electric dipole lagging a quarter of the period behind the latter. Now, if the field radiated by the electric dipole *E_P_*(*t*) is proportional to the second time derivative of 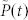, *E_P_*(*t*) ~ i*ωρ*_0_ e^i*ωt*^, the field radiated by the toroidal dipole is proportional to the third time derivative of^17^ 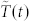, i.e. *E_T_*(*t*) ~−i*ω*^3^*ρ*_0_ e^i*ωt*^. Therefore *E_P_*(*t*) and *E_T_*(*t*) scattered by the dumbbell-shaped aperture are in anti-phase and always interfere destructively (see Supplementary Materials for more details). The amplitude of the poloidal currents increases resonantly when the wavelength of incident radiation *λ* = 2π *c*/*ω* becomes close to the circumference of the aperture ~4π *R* (where *R* is the radius of the circular cuts) leading to the enhancement of toroidal emission, which in principle can be made to cancel electric dipolar scattering completely.

**Figure 2:**
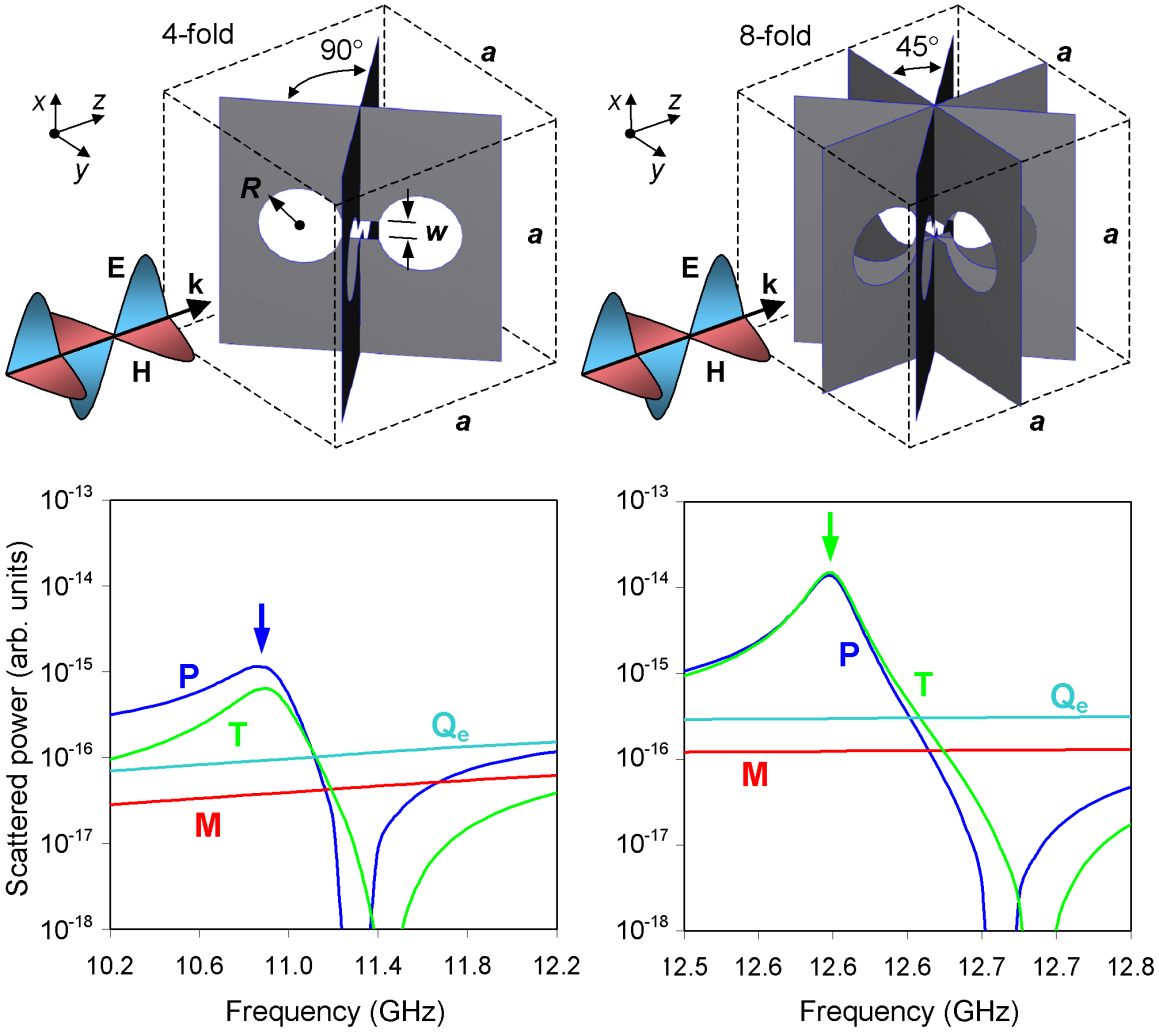
Multipole excitations in toroidal metamaterials. Panels (a) and (b) show metamaterial's unit cell with 4-fold (a) and 8-fold (c) symmetry; *a* = 16.5 mm, *R* = 2.5 mm, *w* = 1.2 mm, separation between the centers of the circular cuts is 7.0 mm. Panels (c) and (d) show dispersions of multipolar scattering rates calculated for 4 strongest multipoles induced in 4-fold (c) and 8-fold (d) symmetric metamolecules. Arrows indicate locations of the corresponding transparency resonances.

In reality, the incident electromagnetic wave induces in such a structure higher oscillating multipoles as well, most notably magnetic quadrupole **Q^(m)^**, which also results from the pair of counter-rotating currents. The scattering contribution from this multipolar current mode can be effectively suppressed in an aperture-based structure of higher rotational symmetry such as, for example, 4-fold or 8-fold symmetric toroidal metamolecules shown in Figs. 2a and 2b. We demonstrated this by modeling numerically the interaction of the metamolecules assembled in two-dimensional arrays (slabs of toroidal metamaterials) with a normally incident linearly polarized plane wave. Calculated densities of the induced electrical currents were used to compute scattered powers of the conventional multipoles and toroidal dipole associated with each metamolecule (see Supplementary Materials for details).

**Figure 3:**
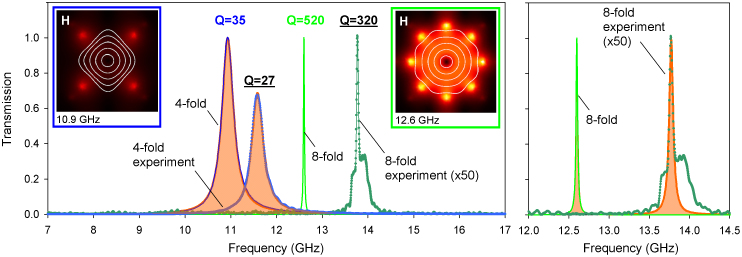
Transmission response of toroidal metamaterials. (a) Blue and green solid curves show simulated spectra of metamaterial slabs based on 4-fold and 8-fold symmetric metamolecules respectively, dots - experimentally measured data (multiplied by the factor of 50 in the case of 8-fold symmetric structure). Orange-filled areas represent symmetric Lorentz line profiles fitting simulation and experimental data for the 4-fold symmetric structure. Insets show simulated distribution of magnetic field lines and magnetic field intensity |**H**| corresponding to resonantly excited modes in 4-fold (left) and 8-fold (right) symmetric metamolecules. (b) Simulated (solid line) and measured (dots) spectra of 8-fold symmetric metamaterial fitted by symmetric Lorentz line profiles (orange-filled areas).

Our calculations showed that, for example, in the metamaterial with 4-fold symmetric metamolecules the emission of the standard multipoles other than electric dipole **P** is small: close to the resonance (which is at around 11 GHz for a centimetre-sized metamolecule) electric quadrupole **Q^(e)^** and magnetic dipole **M** exhibit scattering rates that are factors of 10 and 30 smaller than that of **P**, while **Q^(m)^** is a factor of 10^5^ weaker here (see Fig. 1c). Toroidal dipole provides the second strongest contribution at the resonance being responsible for more than 30% of the total scattering. Its presence can be detected in the near-field as closed loops of magnetic field-lines confined within the metamolecule and threading the circular sections of the apertures (see left inset to Fig. 3).

Our calculations also show that at the resonance the complete destructive interference of radiated fields take place in a loss-less toroidal metamaterial (metals can be treated loss-less in the microwave part of the spectrum). This corresponds to the total transparency of the toroidal metamaterial. For instance, for the 4-fold symmetric system the prevailing electric dipolar scattering is cancelled on about 65% by toroidal scattering and on 35% by scattering from other multipoles (see Supplementary Materials for more details) creating a background-free resonance transparency peak with symmetric Lorenzian profile and *Q* = 35, which is already quite high by the standard of the microwave metamaterials (see Fig. 3). In the metamaterial composed of 8-fold symmetric metamolecules electric and toroidal dipolar scattering is mutually cancelled on 96%, leading to a transparency peak with *Q* ~ 520. The increased role of toroidal dipolar mode in the 8-fold symmetric structure is also evident from the calculated magnetic near-field map (compare the insets to Fig. 3).

We confirmed the appearance of the transparency resonances due to electric-toroidal dipolar interference with experiments conducted in the microwave part of the spectrum. For that we constructed toroidal metamaterials from strips of thin stainless steel plates with dumbbell-shaped apertures, which were assembled into 4- or 8-fold structures (see Fig. 1c). The experimental data shows a very good qualitative agreement with the theory reproducing fully the main features of the metamaterials' response predicted by the modelling: the appearance of narrow isolated bands of transparency with symmetric Lorentzian profile and very large Q-factor, which reaches a record-high value of 320 in the case of 8-fold symmetric metamaterial (see Fig. 3). The slight shift of the observed resonances towards higher frequencies is attributed to the shortening of the outer effective circumference of the apertures due to the non-zero thickness of the metal strips. For the strip thickness of 0.8 mm our estimate gives the relative shortening and correspondingly blue shifts of about 5% and 14% for 4- and 8-fold symmetric metamolecules respectively, which agrees well with the results of the actual measurements (see Supplementary Materials for details). Somewhat lower Q-factors measured for the toroidal metamaterials result from the inhomogeneous broadening of the resonances due to fabrication tolerances and imperfections of the sample assemblies. The latter is also responsible for the incomplete transparency, as well as the appearance of the broad asymmetric pedestal of the high-fidelity resonance. For example, given 0.2% variation of the resonance frequency among 8-fold symmetric metamolecules in the assembled array (which correspond to the fabrication tolerance of 0.06 mm), the amplitude of its transmission resonance would reduce to 4% with the Q-factor dropping to about 300. Other factors that limited the level of transparency in the experiments are residual curvature of the incident wavefront and diffusive scattering from the metamaterial structures.

## Discussion

We point out that simultaneous presence of collocated electric and toroidal dipolar excitations in our metamaterials may create a unique situation, where vector potential **A** generated outside the arrays is non-zero even though the re-emitted (in particular, reflected) electromagnetic fields are completely cancelled via the destructive interference. Indeed, although the electromagnetic field emission characteristics of oscillating 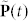 and 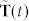 are identical, the vector-potential fields they produce are quite different^17^,^20^. Under the condition of total coherent cancellation of their radiation the resulting vector potential propagating in the far-field is given by 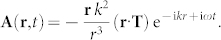


The complete expression for **A** featuring also the near-field contributions can be found in Supplementary Materials.

The discussion on the independent physical significance of the vector potential started from the famous paper by Aharonov and Bohm^21^ and has developed into an active research topic that attracted a large number of publications (see, for example^22^,^23^,^24^ and references therein). In particular, it was theoretically shown that the Aharonov-Bohm effect due to time-varying vector potential of a coherent light source could be used for quantum-nondemolition detection of photons emitted by the source^25^. Intriguingly, Afanasiev and Stepanovsky suggested in their paper^17^ that time-dependent electromagnetic potentials produced by their non-trivial non-radiating system of collocated electric and toroidal dipolar excitations “… can be used as a new channel for information transfer (by modulating the phase of the charged-partial wavefunction) and for the performance of time-dependent Aharonov-Bohm-like experiments.”. Those predictions were disputed by Marengo and Ziolkowski, who argued “… that electrodynamically not only the fields but also the associated potentials are unobservable everywhere in the exterior of a spatially localized non-radiating source” and “… Aharonov-Bohm effects associated with non-radiating potentials are possible only in static situations.”^26^. Resolving this apparent controversy and further understanding the role of time-dependent electromagnetic potentials would require practical realization of non-trivial non-radiating sources, which have so far remained merely a peculiar theoretical concept.

We believe that the observed resonant transparency phenomenon is the first example of the manifestation of the non-trivial non-radiating charge-current excitation, while toroidal metamaterial arrays operating at the transparency resonance could serve as the source of the non-compensated vector-potential. Even though in our case the destructive interference between electric and toroidal dipole moments does not lead to the complete cancellation of their scattering, the residual dipolar radiation (which converges in forward and backward directions for an infinite array of sub-wavelength periodicity) is suppressed by higher multipoles, while the gauge-invariant vector-potential should be generated by the mutually compensated contributions of **P** and **T**. Here, due to the two-dimensional periodicity of the metamaterial arrays, the non-trivial component of the vector-potential field is expected to localize in the plane of the metamaterial structure.

In conclusion, we have identified a class of metamaterials supporting a novel mechanism of resonant electromagnetic transparency, which is different from Fano interference typically encountered in the plasmonic and metamaterial systems mimicking electromagnetically-induced transparency. It exploits destructive interference between spatially and spectrally collocated and coherently oscillating induced electric and toroidal dipoles, and produces very narrow and characteristically symmetric Lorentzian transparency lines with Q-factors exceeding 300. Such mode of the metamaterial resonant excitation corresponds to a long-awaited implementation of the non-trivial non-radiating charge-current configuration that might generate waves of gauge-irreducible vector potential in the absence of scattered (reflected) electromagnetic fields.

## Methods

### Numerical modelling

The metamaterial slabs were described through periodic boundary conditions applied to the corresponding unit cell's facets in X and Y directions, as indicated in Figs. 2a and 2b. The dumbbell-shaped aperture resonators forming 4-fold and 8-fold symmetric toroidal metamolecules were assumed to be cut in infinitely thin sheets of perfect electric conductor in accordance with the design specifications. Electromagnetic response of the metamaterial slabs was simulated using commercial full three-dimensional Maxwell equations solver based on the finite element method, COMSOL 3.5a. The simulations also provided data on the densities of electrical currents induced in the metamaterials by the incident wave, which was used to compute scattered powers of the conventional multipoles and toroidal dipole associated with each metamolecule.

### Samples fabrication

Metamaterial slabs were constructed from strips of thin stainless steel plates with dumbbell-shaped apertures. The stainless steel strips, which had the thickness of 0.8 mm, were patterned using chemical etching and assembled into columns of 4-fold and 8-fold symmetric structures. The metamaterial slabs were formed by 15 such columns, which contained 16 toroidal metamolecules each (see Fig. 1c). All dimensions of the design features were identical to those used in our modelling (Fig. 2).

### Microwave measurements

Transmission spectra of the metamaterial samples were measured in the Emerson & Cuming microwave anechoic chamber using vector network analyser (Agilent E8364B) and a pair of broadband linearly polarized horn antennas (Schwarzbeck Mess-Elektronik BBHA 9120D) equipped with dielectric lens collimators.

## Author Contributions

The idea of the research was conceived by N.I.Z. and V.A.F.; the metamaterial structure was proposed, manufactured and experimentally measured by V.A.F.; computer simulations were performed by A.V.R. and V.S.; V.A.F., N.I.Z., A.V.R., V.S. and D.P.T. discussed the results and analysed the data; N.I.Z., V.A.F. and D.P.T. wrote the paper.

## Supplementary Material

Supplementary Information Supplementary Material

## References

[b1] Jackson J. D. [Chapter 16] Classical Electrodynamics [3rd edition]. (Wiley, New York, 1999).

[b2] Landau L. D. & Lifschitz E. M. Course of Theoretical Physics. vol. 2, [4th revised English edition] (Pergamon Press, New York, 1993).

[b3] Dubovik V. M. & Cheshkov A. A. Multipole expansion in classical and quantum field theory and radiation. Sov. J. Part. Nucl. Phys. 5, 318–364 (1974).

[b4] Afanasiev G. N. & Dubovik V. M. Electromagnetic properties of a toroidal solenoid. J. Phys. A Math. Gen. 25, 4869–4886 (1992).

[b5] Gongora T. A. & Ley-Koo E. Complete electromagnetic multipole expansion including toroidal moments. Rev. Mex. Fis. E 52, 188–197 (2006).

[b6] Dubovik V. M. & Tugushev V. V. Toroidmoments in electrodynamics and solid-state physics. Phys. Rep. 187, 145–202 (1990).

[b7] Radescu E. E. & Vaman G. Exact calculation of the angular momentum loss, recoil force, and radiation intensity for an arbitrary source in terms of electric, magnetic, and toroid multipoles. Phys. Rev. E 65, 046609 (2002). 10.1103/PhysRevE.65.046609 12006049

[b8] Haxton W. C. Atomic parity violation and the nuclear anapole moment. Science 275, 1753 (1997).

[b9] Hasty R. *et al.* Science 290, 2117–2119 (2000). 11118140 10.1126/science.290.5499.2117

[b10] Ceulemans A., Chibotaru L. F. & Fowler P. W. Molecular Anapole Moments. Phys. Rev. Lett. 80, 1861–1864 (1998).

[b11] Naumov I. I., Bellaiche L. & Fu H. Unusual phase transitions in ferroelectric nanodisks and nanorods. Nature 432, 737–740 (2004). 15592408 10.1038/nature03107

[b12] Kaelberer T., Fedotov V. A., Papasimakis N., Tsai D. P. & Zheludev N. I. Toroidal dipolar response in a metamaterial. Science 330, 1510–1512 (2010). 21051597 10.1126/science.1197172

[b13] Huang Y.-W. *et al.* Design of plasmonic toroidal metamaterials at optical frequencies. Opt. Express 20, 1760–1768 (2012). 22274519 10.1364/OE.20.001760

[b14] Guo L. Y. *et al.* Electric toroidal dipole response in split-ring resonator metamaterials. Eur. Phys. J. B 85, 208 (2012).

[b15] Dong Z. G. *et al.* Toroidal dipole response in a multifold double-ring metamaterial. Opt. Express 20, 13065–13070 (2012). 22714333 10.1364/OE.20.013065

[b16] Ogut B., Talebi N., Vogelgesang R., Sigle W. & van Aken P. A. Toroidal plasmonic eigenmodes in oligomer nanocavities for the visible. Nano Lett. 12, 5239–5244 (2012) 22935079 10.1021/nl302418n

[b17] Afanasiev G. N. & Stepanovsky Y. P. The electromagnetic field of elementary time-dependent toroidal sources. J. Phys. A Math. Gen. 28, 4565–4580 (1995).

[b18] Boardman A. D., Marinov K., Zheludev N. & Fedotov V. A. Dispersion properties of nonradiating configurations: Finite-difference time-domain modeling. Phys. Rev. E 72, 036603 (2005). 10.1103/PhysRevE.72.036603 16241588

[b19] Luk'yanchuk B. *et al.* The Fano resonance in plasmonic nanostructures and metamaterials. Nat. Mater. 9, 707–715 (2010). 20733610 10.1038/nmat2810

[b20] Afanasiev G. N. & Dubovik V. M. Some remarkable charge-current configurations. Phys. Part. Nuclei 29, 366–391 (1998).

[b21] Aharonov Y. & Bohm D. Significance of electromagnetic potentials in the quantum theory. Phys. Rev. 115, 485–491 (1959).

[b22] Tonomura A. & Nori F. Quantum physics: Disturbance without the force. Nature 452, 298–299 (2008). 18354473 10.1038/452298a

[b23] Batelaan H. & Tonomura A. The Aharonov–Bohm effects: variations on a subtle theme. Phys. Today 62, 38 (2009).

[b24] Zaric S. *et al.* Optical signatures of the Aharonov-Bohm phase in single-walled carbon nanotubes. Science 304, 1129–1131 (2004). 15155942 10.1126/science.1096524

[b25] Lee B., Yin E., Gustafson T. K. & Chiao R. Analysis of Aharonov-Bohm effect due to time-dependent vector potentials. Phys. Rev. A 45, 4319–4325 (1992). 9907506 10.1103/physreva.45.4319

[b26] Marengo E. A. & Ziolkowski R. W. Nonradiating sources, the Aharonov-Bohm effect, and the question of measurability of electromagnetic potentials. Radio Sci. 37, 19 (2002).

