# Supplementary material for: Resonant Transparency and Non-Trivial Non-Radiating Excitations in Toroidal Metamaterials
Source: Sci Rep. 2013 Oct 17;3:2967. doi: 10.1038/srep02967 (PMC3797985; doi:10.1038/srep02967)
Supplement: Supplementary Information — Supplementary Material [file srep02967-s1.pdf]

Supplementary Material for

Resonant Transparency and Non-Trivial Excitations in Toroidal Metamaterials

V. A. Fedotov[1], A. V. Rogacheva[1], V. Savinov[1], D. P. Tsai[2,3], N. I. Zheludev[1, 4]

[1]*Optoelectronics Research Centre and Centre for Photonic Metamaterials, University of Southampton, SO17 1BJ, UK*
[2]*Department of Physics, National Taiwan University, Taipei 10617, Taiwan*
[3]*Research Center for Applied Sciences, Academia Sinica, Taipei 115, Taiwan*
[4]*Centre for Disruptive Photonics Technologies, Nanyang Technological University, 637371 Singapore*

Evaluation of Power scattered by multipoles

The relative strength of the induced multipole moments contributing to the far-field response of the metamaterial was evaluated using expression for the total radiated power derived by Radescu & Vaman [2], to the 5th order of (1/c). Note that CGS units are used. In case of harmonic excitation ~exp(iωt) the expression for the radiated power has the form:

$$I = \frac{2\omega^4}{3c^3}|\mathbf{P}|^2 + \frac{2\omega^4}{3c^3}|\mathbf{M}|^2 + \frac{4\omega^5}{3c^4}\mathrm{Im}\left(\mathbf{P}^\dagger\mathbf{T}\right) + \frac{2\omega^6}{3c^5}|\mathbf{T}|^2 + \frac{\omega^6}{5c^5}\left|\mathbf{Q}^{(e)}\right|^2 + \\ + \frac{\omega^6}{20c^5}\left|\mathbf{Q}^{(m)}\right|^2 - \frac{2\omega^6}{15c^5}\mathrm{Re}\left(\mathbf{M}^\dagger\langle\mathbf{R}_m^2\rangle\right) \quad (1)$$

Where, for the quadrupoles, we use the notation $|\mathbf{Q}|^2 = \Sigma_{\alpha,\beta = x,y,z}|\mathrm{Q}_{\alpha\beta}|^2$, the complex conjugation is denoted by the 'dagger' symbol ($\mathbf{P}^\dagger$).

The first two terms correspond to the conventional electric (charge) and magnetic dipole scattering. The third term accounts for the interference between the electric and toroidal dipoles, which is generally non-zero because both types of dipoles have the same angular momentum and parity properties. The fourth term corresponds to the toroidal dipole scattering. The fifth and sixth terms come from electric and magnetic quadrupoles. The last term is a further correction resulting from the finite size of the current distribution in the meta-molecule. It is known as the "mean-square radius of the magnetic dipole term" and arises as a result of higher order Taylor-expansion terms of the spherical Bessel functions (the conventional multipoles being the zeroth-order terms). For the purpose of our calculations we used Cartesian basis ($\alpha,\beta = x,y,z$) and the following representations of the multipoles and mean-square radius of the magnetic dipole distribution [2]:

$$\mathrm{P}_\alpha = \frac{1}{\mathrm{i}\omega}\int d^3r\ \mathrm{J}_\alpha$$

$$\mathrm{M}_\alpha = \frac{1}{2c}\int d^3r\ [\mathbf{r}\times\mathbf{J}]_\alpha$$

$$\mathrm{T}_\alpha = \frac{1}{10c}\int d^3r\ [(\mathbf{r}\cdot\mathbf{J})\ \mathrm{r}_\alpha - 2\mathrm{r}^2\mathrm{J}_\alpha]$$

$$\langle\mathrm{R}_m^2\rangle_\alpha = \frac{1}{2c}\int d^3r\ [\mathbf{r}\times\mathbf{J}]_\alpha\, r^2$$

$$\mathrm{Q}^{(e)}{}_{\alpha\beta} = \frac{1}{\mathrm{i}2\omega}\int d^3r\left[\mathrm{r}_\alpha\,\mathrm{J}_\beta + \mathrm{J}_\alpha\mathrm{r}_\beta - \frac{2}{3}\delta_{\alpha\beta}(\mathbf{r}\cdot\mathbf{J})\right]$$

$$\mathrm{Q}^{(m)}{}_{\alpha\beta} = \frac{1}{3c}\int d^3r\ \left[[\mathbf{r}\times\mathbf{J}]_\alpha\ \mathrm{r}_\beta + \mathrm{r}_\alpha\ [\mathbf{r}\times\mathbf{J}]_\beta\right]$$

We used the charge conservation relation $\mathrm{i}\omega\rho + \nabla\cdot\mathbf{J} = 0$, to eliminate the charge density (ρ) in favour of current density ($\mathbf{J}$) in the electric dipole and quadrupole.

Multipolar contributions to the metamaterial scattering at its transparency resonance

Here we show how various multipole moments resonantly induced in the 4-fold symmetric metamolecules by the incident plane wave contribute to the electromagnetic scattering of the entire metamaterial array. The multipole moments were obtained from the current density extracted from the numerical simulations of the metamaterial response (COMSOL 3.5a). The multipolar field contributions are presented in Fig. 1 in the form of a phasor diagram, which contains both the amplitude and phase of the scattered (i.e. reflected) electromagnetic waves calculated for the frequency of the metamaterial transmission resonance $\nu = 11.09$ GHz. The scattered fields produced by the electric, **P**, and toroidal, **T**, dipoles have the largest amplitudes and oscillate in anti-phase with **P** cancelled by **T** on about 65 %. The scattering from electric quadrupole, $\mathbf{Q}^{(e)}$, and magnetic dipole, **M**, is small but nevertheless important: the fields they produce oscillate in phase with the field of the toroidal dipole moment, and thus compensate the remaining 35 % of electric dipole scattering leading to the total electromagnetic transparency (zero reflection). The multipolar contributions to the plane-wave scattering were calculated by summing the fields of the respective multipole moments arranged in an infinite two-dimensional lattice [3].

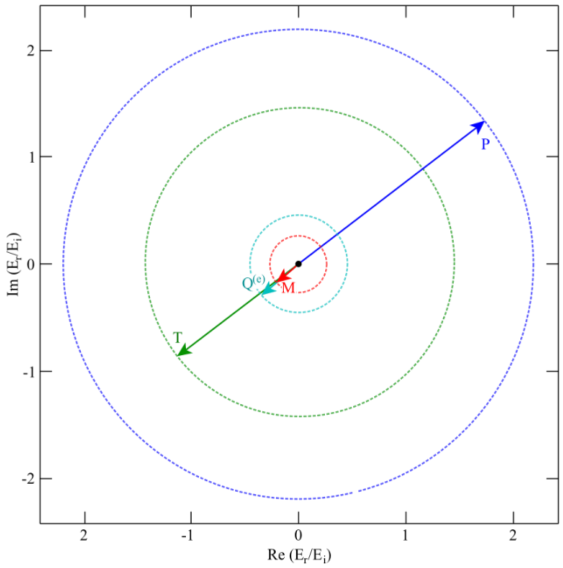

Figure S1. Complete electromagnetic transparency. The total amplitude of the waves scattered (reflected) by the metamaterial array at υ=11.09 GHz due to various multipoles induced in the 4-fold symmetric metamolecules is zero. The data is presented in terms of real and imaginary parts of the reflected fields E_r, normalized with respect to the incident field E_i.

Evaluation of the electro-magnetic fields and the vector potential scattered by electric and toroidal dipoles

In our discussion we refer to the interference between the electric and the toroidal dipoles and the residual vector potential field. Here, following Afanasiev and Stepanovsky [1], we give the full distribution of the electromagnetic fields (**E, H**) as well as the scalar φ and vector potential **A**, emitted by both types of multipoles. We specify all three types of radiated fields in terms of multipole moments. Note that CGS units are used. For the electric dipole $\tilde{\mathbf{P}}(t) = \mathbf{P}e^{i\omega t}$ the radiated fields are:

$$\varphi_P = \frac{(\mathbf{r}\cdot\mathbf{P})D(\omega,r)}{cr}\,\frac{\exp(-\mathrm{i}kr+\mathrm{i}\omega t)}{r}, \qquad \mathbf{A}_P = \mathrm{i}k\,\mathbf{P}\,\frac{\exp(-\mathrm{i}kr+\mathrm{i}\omega t)}{r}$$

$$\mathbf{E}_P = \left[\frac{(\mathbf{r}\cdot\mathbf{P})F(\omega,r)}{c^2r^2}\mathbf{r} - \frac{G(\omega,r)}{c^2}\mathbf{P}\right]\frac{\exp(-\mathrm{i}kr+\mathrm{i}\omega t)}{r}$$

$$\mathbf{H}_P = -\frac{\mathrm{i}kD(\omega,r)}{cr}\,[\mathbf{r}\times\mathbf{P}]\,\frac{\exp(-\mathrm{i}kr+\mathrm{i}\omega t)}{r}$$

For the toroidal dipole $\tilde{\mathbf{T}}(t) = \mathbf{T}e^{i\omega t}$:

$$\varphi_T = 0, \qquad \mathbf{A}_T = \left[\frac{(\mathbf{r}\cdot\mathbf{T})F(\omega,r)}{c^2r^2}\mathbf{r} - \frac{G(\omega,r)}{c^2}\mathbf{T}\right]\frac{\exp(-\mathrm{i}kr+\mathrm{i}\omega t)}{r}$$
$$\mathbf{E}_T = \left[\frac{\mathrm{i}kG(\omega,r)}{c^2}\mathbf{T} - \frac{\mathrm{i}k(\mathbf{r}\cdot\mathbf{T})F(\omega,r)}{c^2r^2}\mathbf{r}\right]\frac{\exp(-\mathrm{i}kr+\mathrm{i}\omega t)}{r}$$
$$\mathbf{H}_T = -\frac{k^2D(\omega,r)}{cr}[\mathbf{r}\times\mathbf{T}]\frac{\exp(-\mathrm{i}kr+\mathrm{i}\omega t)}{r}$$

In both cases we have used the following functions to simplify expressions:

$$D(\omega,r) = \mathrm{i}\omega + \frac{c}{r} = c\left(\mathrm{i}k + \frac{1}{r}\right)$$
$$F(\omega,r) = -\omega^2 + \frac{\mathrm{i}3c\omega}{r} + \frac{3c^2}{r^2} = c^2\left(-k^2 + \frac{\mathrm{i}3k}{r} + \frac{3}{r^2}\right)$$
$$G(\omega,r) = -\omega^2 + \frac{\mathrm{i}c\omega}{r} + \frac{c^2}{r^2} = c^2\left(-k^2 + \frac{\mathrm{i}k}{r} + \frac{1}{r^2}\right)$$

One can see that the electric and magnetic fields radiated by the two multipoles are very similar. We make this similarity explicit by combining the fields of the toroidal and electric dipoles:

$$\mathbf{A}_{tot} = \mathbf{A}_P + \mathbf{A}_T = \left[\frac{(\mathbf{r}\cdot\mathbf{T})F(\omega,r)}{c^2r^2}\mathbf{r} - \frac{G(\omega,r)}{c^2}\mathbf{T} + \mathrm{i}k\mathbf{P}\right]\frac{\exp(-\mathrm{i}kr+\mathrm{i}\omega t)}{r}$$
$$\mathbf{E}_{tot} = \mathbf{E}_P + \mathbf{E}_T = \left[\frac{\big(\mathbf{r}\cdot(\mathbf{P}-\mathrm{i}k\mathbf{T})\big)F(\omega,r)}{c^2r^2}\mathbf{r} - \frac{G(\omega,r)}{c^2r}(\mathbf{P}-\mathrm{i}k\mathbf{T})\right]\frac{\exp(-\mathrm{i}kr+\mathrm{i}\omega t)}{r}$$
$$\mathbf{H}_{tot} = \mathbf{H}_P + \mathbf{H}_T = -\frac{\mathrm{i}kD(\omega,r)}{cr}[\mathbf{r}\times(\mathbf{P}-\mathrm{i}k\mathbf{T})]\frac{\exp(-\mathrm{i}kr+\mathrm{i}\omega t)}{r}$$

If $\mathbf{P} = \mathrm{i}k\mathbf{T}$, the radiation from the electric and the toroidal dipoles interferes destructively and both $\mathbf{E}_{tot}$ and $\mathbf{H}_{tot}$ vanish. Here we presented the full fields radiated by the dipoles, including the near-field, so the destructive interference is absolute (both in the near- and far-field). The vector potential, however, remains non-zero and spreads in the far-field as a spherical wave:

$$\mathbf{A}_{tot}^{(\mathbf{P}=\mathrm{i}k\mathbf{T})} = \left[\frac{(\mathbf{r}\cdot\mathbf{T})F(\omega,r)}{c^2r^2}\mathbf{r} - \frac{D(\omega,r)}{cr}\mathbf{T}\right]\frac{\exp(-\mathrm{i}kr+\mathrm{i}\omega t)}{r}$$

Here we shall note that the intensity of radiation scattered by just the electric and the toroidal dipoles may be written from of Eq. (1) as:

$$I_{P,T} = \frac{2\omega^4}{3c^3}|\mathbf{P}|^2 + \frac{4\omega^5}{3c^4}\mathrm{Im}\big(\mathbf{P}^\dagger\mathbf{T}\big) + \frac{2\omega^6}{3c^5}|\mathbf{T}|^2 = \frac{2ck^4}{3}|\mathbf{P}|^2 + \frac{4ck^5}{3}\mathrm{Im}\big(\mathbf{P}^\dagger\mathbf{T}\big) + \frac{2ck^6}{3}|\mathbf{T}|^2$$

For the absolute destructive interference ($\mathbf{P} = \mathrm{i}k\mathbf{T}$) we confirm that total radiated power of the electric and the toroidal dipoles is vanishing:

$$I_{P,T}^{(\mathbf{P}=\mathrm{i}k\mathbf{T})} = \frac{2ck^6}{3}|\mathbf{T}|^2 + \frac{4ck^5}{3}\mathrm{Im}(-\mathrm{i}k|\mathbf{T}|^2) + \frac{2ck^6}{3}|\mathbf{T}|^2 = \frac{4ck^6}{3}|\mathbf{T}|^2 - \frac{4ck^6}{3}|\mathbf{T}|^2 = 0$$

Explanation of the shift of resonance observed in the experiments

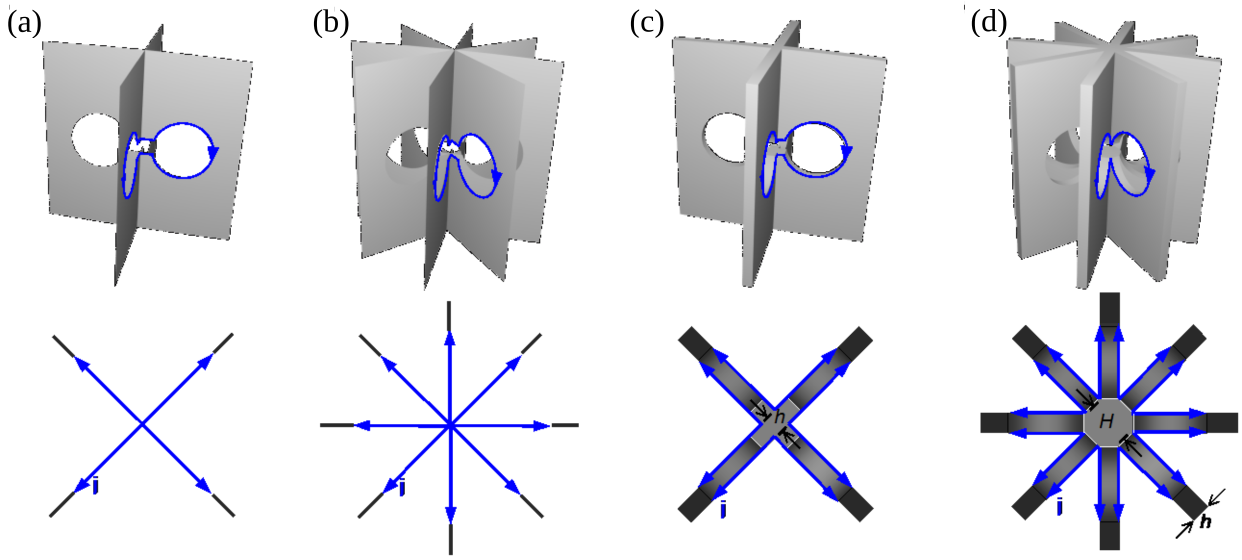

Fig. D1. 4- and 8-fold symmetric toroidal metamolecules and their top-view cross-sections, which are schematically shown for (a, b) modelled structres with dumbbel-shaped apertures made in infinitely thin sheets of metal, and (c, d) fabricated structures assembled from metal strips of thickness *h*. Blue arrows indicate localizations of resonantly induced poloidal-like currents **J** in the apertures of the metamolecules.

Due to non-zero thickness of the metal strips, *h*, the aperture's outer circumference that supports the induced poloidal-like currents **J** (as traced by blue lines in the top panels of Fig. D1) is effectively shortened by $2h$ in 4-fold structure, and by $2H$ in 8-fold structure (see Fig. D1). It is easy to show that $H = 5h/4$. Given that $h = 0.8$ mm the outer circumference of the aperture, which is ~ 33 mm for the case of infinitely thin strips, is reduced by about 1.05 and 1.14 times in correspondingly 4- and 8-fold symmetric structures. That translates into 5% and 14% increase of their resonance frequencies.

References

1 G. N. Afanasiev and Y. P. Stepanovsky, J. Phys. A Math. Gen. 28, 4565 (1995).
2 E. E. Radescu and G. Vaman, Phys. Rev. E 65, 046609 (2002).
3 V. Savinov, V. A. Fedotov, and N. I. Zheludev, arXiv:1310.0106v1 (2013).
